# Vocal imitations and production learning by Australian musk ducks (*Biziura lobata*)

**DOI:** 10.1098/rstb.2020.0243

**Published:** 2021-10-25

**Authors:** Carel ten Cate, Peter J. Fullagar

**Affiliations:** ^1^ Institute of Biology, Leiden University, PO Box 9505, 2300 RA Leiden, The Netherlands; ^2^ Unit 1/11 Joy Cummings Place, Belconnen, ACT 2617, Australia

**Keywords:** vocal imitation, vocal learning, Australian musk duck, birds, waterfowl

## Abstract

Acquiring vocalizations by learning them from other individuals is only known from a limited number of animal groups. For birds, oscine and some suboscine songbirds, parrots and hummingbirds demonstrate this ability. Here, we provide evidence for vocal learning in a member of a basal clade of the avian phylogeny: the Australian musk duck (*Biziura lobata*). A hand-reared individual imitated a slamming door and a human voice, and a female-reared individual imitated Pacific black duck quacks. These sounds have been described before, but were never analysed in any detail and went so far unnoticed by researchers of vocal learning. The imitations were produced during the males' advertising display. The hand-reared male used at least three different vocalizations in the display context, with each one produced in the same stereotyped and repetitive structure as the normal display sounds. Sounds of different origins could be combined in one vocalization and at least some of the imitations were memorized at an early age, well before they were produced later in life. Together with earlier observations of vocal differences between populations and deviant vocalizations in captive-reared individuals, these observations demonstrate the presence of advanced vocal learning at a level comparable to that of songbirds and parrots. We discuss the rearing conditions that may have given rise to the imitations and suggest that the structure of the duck vocalizations indicates a quite sophisticated and flexible control over the vocal production mechanism. The observations support the hypothesis that vocal learning in birds evolved in several groups independently rather than evolving once with several losses.

This article is part of the theme issue ‘Vocal learning in animals and humans’.

## 1. Introduction

Vocal production learning, i.e. acquiring vocalizations through learning, is a crucial component of human speech and language development. The extent of the human ability to imitate and adjust vocalizations based on auditory experience or some form of feedback shows a major difference with the vocal learning abilities of other primate species, including the great apes. There is ample evidence that primates of various species raised in social isolation or cross-fostered to other species develop largely normal species-specific vocalizations (e.g. [1,2]) and modifications and deviations from normal species-specific vocalizations seem only possible within relatively tight species-specific constraints [2]. However, vocal learning has evolved in several other animal groups, in which it may have arisen independently. This independent occurrence provides important opportunities for comparative studies on mechanisms, function and evolution of vocal learning, which may also shed light on the mechanisms and evolution of vocal learning in our own species.

Definitions of vocal production learning vary among researchers and, along with that, the type of vocal modifications that are considered evidence of vocal learning (e.g. [2–6]). Nevertheless, there is agreement that strongly deviating vocalizations in animals reared in isolation, the imitation of sounds of other species, and/or the presence and imitation of individually distinct vocal variants within a species demonstrate the presence of vocal learning. Among mammalian groups, such undisputed evidence is present for several whales, dolphins and pinnipeds [7], bat species [8] and elephants [9]. Among birds, oscine and some suboscine songbirds, parrots and hummingbirds all provide examples of vocal learning by several of the above-mentioned criteria. Reviewing the evidence for different bird groups suggests that some form of vocal learning may also be present in some other bird groups; however, the extent of learning is usually limited and clear examples remain rare [10]. Strong evidence for the presence of vocal learning are imitations of human speech or mechanical sounds, as observed in several songbird [11–14] and parrot species [14–17], in particular when held or raised in captivity. The only reports of imitation of speech and human-made sounds outside songbirds and parrots, and hence strong indication of the presence of vocal learning, concern a hand-reared captive Australian musk duck (*Biziura lobata*), ‘Ripper’, imitating a slamming door and producing some speech-like sounds [18–20]. In addition, another musk duck was reported to imitate a Pacific black duck (*Anas superciliosa*) [20].

The musk duck is an endemic Australian species. It belongs to the order of Anseriformes (waterfowl). Together with the Galliformes (land fowl), the Anseriformes are grouped as the Galloanseres, considered to be a basal clade in the avian phylogeny, separated from all other avian orders for about 90 Myr [21]. Despite the long history of the domestication of various duck and goose species and the frequent incidence of hand-raised or cross-fostered individuals, there has been no report or even suggestion that this clade might contain any species showing vocal learning. Because of the large phylogenetic gap separating Anseriformes from all other clades showing any form of vocal learning and the lack of evidence for vocal learning in other Anseriformes, vocal learning in the musk duck would represent a case of independent evolution, raising many questions ranging from the neural and behavioural mechanisms involved to the evolutionary and adaptive background of vocal learning in this species. Therefore, the reported imitations call for a more extensive documentation and analysis. The various reports on the imitating musk ducks are all based on recordings by PJ Fullagar in 1987 and 2000. Here, we provide the available information concerning these recordings. We analyse the sounds produced, describe the context in which they occurred and relate this to the normal sound repertoire. We discuss what the findings tell about the learning and production mechanism(s) that may have been involved and how these relate to those observed in other taxa.

### (a) Musk ducks, behaviour and vocalizations

Musk ducks are distributed over two separated geographic regions in western and southeast Australia, respectively [20]. They are heavy-bodied short-winged grey coloured ducks that show extreme sexual dimorphism with adult males up to three times larger than females. They have short legs but large feet and dive readily but rarely fly. Adult males have a large pendulous lobe hanging below the bill that can be flacid or turgid. A pungent musky odour that disipates rapidly can be detected from dominant males but not from all males and not from females. Male musk ducks are promiscuous with dominant males displaying at leks to attract females that nest well away from them. Unlike other waterfowl, females conceal their downy young until well grown and feed them (see [18,22,23]). Musk ducks have rarely been bred in captivity, largely due to the difficulty of managing the aggressive mature males that are prone to attacking other waterfowl.

**Figure 1.**
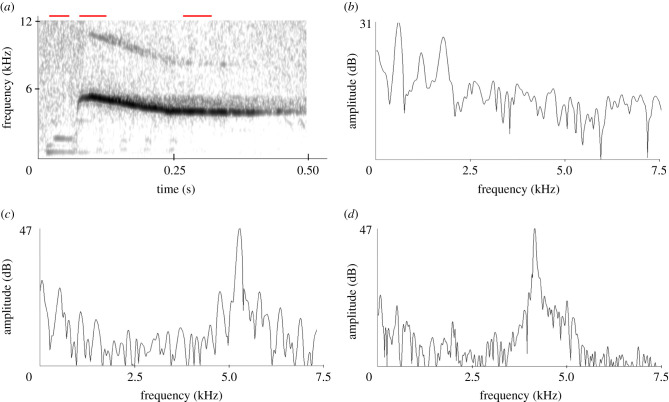
(*a*) Sonogram of a high-pitched whistle-kick of a wild musk duck, showing the soft sound consisting of three harmonics followed by the loud whistle. Red lines indicate origins of power spectra. Recorded by PJ Fullagar at Lake Tongo NSW, 1982 with a Sony Walkman professional cassette recorder and Sennheiser MKH 816 microphone. (*b*) Power spectrum of the soft sound indicating a fundamental of 596 Hz. (*c*) Power spectrum of the start of the whistle. The low-frequency peaks correspond to the same frequencies as in (*b*), the high amplitude peak is at 5.18 kHz. (*d*) Power spectrum of the final part of the whistle, with a peak at 4.08 kHz.

Male display has been described in detail [19,20,22] and involves contorted body actions including the raising and lowering of the large stiff tail accompanied by loud splashing from violent sideways and backwards kicks of the feet. The head is tilted backwards with feathers raised and the large turgid lobe is flaunted. The two musk duck populations differ consistently in their display characteristics and in particular in the sounds accompanying them [20]. Ripper and the second musk duck belonged to the south-eastern population. Male musk ducks from this population perform three display components, corresponding to three escalating levels of intensity [20,22]: (i) a non-vocal display called the paddle-kick, (ii) a second non-vocal display, the plonk-kick, and (iii) a vocal display, called the whistle-kick. In all displays, the feet are used to kick water, while the tail is kept in different positions during the different displays. The whistle-kick consists of a non-vocal splash component produced by the feet hitting the water, followed by two distinct vocal components: a soft low-frequency sound followed by a much louder whistle. There are two variants of this whistle-kick. The vocal part of the high-pitched whistle-kick (figure 1*a,b*) is a soft percussion-like sound with three harmonics, followed by a higher pitched decrescendo concave up-shaped whistle with an average initial frequency of about 5.2 kHz, ending at about 3.7 kHz [20]. In the low-pitched whistle-kick, the percussion sound lacks harmonics and the whistle is a concave down shape from about 5.1 to 2.6 kHz [20]. Immature individuals produce variable and incomplete whistle-kicks, suggesting a gradual development of the sound component, while captive-reared birds often produce sounds considerably different from wild birds [20].

## 2. Methods

### (a) Rearing and recording of the musk ducks

Ripper was a male musk duck captive-reared at Tidbinbilla Nature Reserve, located about 50 km SW of Canberra, Australia. Unfortunately, all documents from Tidbinbilla were lost in the wildfire that swept through the reserve in January 2003 making it difficult to establish all the exact details. Ripper was raised from a fresh egg sourced from East Gippsland, Victoria, Australia in September 1983 and was the only musk duck present at the time of rearing. It was hatched under a foster bantam hen and then raised and fed by hand without the foster hen. After hatching, ‘Ripper’, as he was named, was transferred to an improvised rearing structure created by modifying a stainless steel wash-down table with a sink filled with water and a dry area with a heat lamp above it. After a few weeks, Ripper was most likely first transferred to a small pond with various other captive-reared waterfowl present. Sometime later he was moved to a small pen surrounded by dense shrubbery and concealed from public view. This structure was divided into two halves, with connecting holes below water level that were big enough for a female but too small for Ripper to pass through. Additional musk ducks, initially two females, were obtained from Serendip Reserve, Victoria, sometime before the date of the sound recording of Ripper, and these were almost certainly present in the adjacent part of the pen when the mimicry sound recording was made.

The recordings of Ripper were made using a Sony Walkman Professional cassette recorder and a Sennheiser MKH 816 microphone on July 19 and 26, 1987, at which time Ripper was 4 years old. The recordings consist of three sequences of repeated vocalizations, each containing a different type of vocal imitation. These recordings are present at the Australian National Wildlife Collection as files X49184 and X49185, but our analyses and the examples provided as electronic supplementary material are based on PJ Fullagar's own files.

The recording of the second duck is from another male raised by a captive female with free access to a large pond at Tidbinbilla. He was recorded in June 2000, using a Sony TCD-10 PRO DAT recorder at a distance of about 5 m. This duck must have been 2–3 years old at the time of recording, although this cannot be confirmed because of the above-mentioned loss of records due to the fire at Tidbinbilla.

### (b) Acoustic analysis

The recordings were analysed using the Praat software v. 5.4.12 (www.praat.org). The settings for broadband sonograms were FFT with 1000 time and 250 frequency steps, 0.005 s window length and Gaussian window. The settings for narrowband sonograms were the same, but used a window length of 0.03 s. Power spectra were prepared using Fast Fourier Transform.

## 3. Results

### (a) Ripper

The vocal imitations produced by Ripper were shown in combination with the posture of the ‘whistle-kick’ display. However, the display lacked both the kicks with the feet as well as the vocal components that normally accompany the posture. Instead, three types of imitations were recorded, which we describe in more detail below: (i) slamming door; (ii) slamming door followed by speech-like mumble; and (iii) speech-like phrase that can be described as ‘you bloody foo(l)(d)’. All of these sounds were given in a series of repetitions, similar to the normal whistle-kick display, which is also repetitive.

#### (i) Slamming door imitation

**Figure 2.**
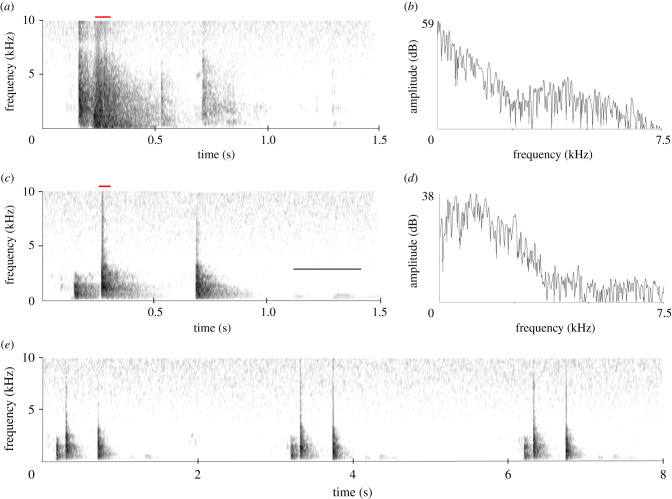
(*a*) Sonogram and (*b*) power spectrum of opening and closing of a metal aviary door (electronic supplementary material, SI 1). (*c*) Sonogram and (*d*) power spectrum of Ripper's imitation (electronic supplementary material, SI 2). Red lines indicate origins of power spectra. Black line in (*c*) indicates a soft ‘bwoo’ sound. (*e*) Three subsequent imitating vocalizations showing the stereotyped nature and regularity in interval duration.

Figure 2*a* shows a sonogram of the opening and closing of a double-hung spring door located between 2 m and 3 m from the sink in which Ripper was kept in the first weeks after hatching. The full succession of opening and closing produced three sound elements (electronic supplementary material, SI 1). This door was used frequently during the day and the hinge springs emitted a distinctive ‘Whuk whuk’ or ‘Whuk whuk wh’ depending on how many times the door oscillated before coming to rest. Figure 2*b* gives a power spectrum of one of the three elements, showing the noisy nature of the sound. Figure 2*c* shows the sonogram of the vocalization produced by Ripper, recorded at about 2–4 m distance (electronic supplementary material, SI 2). The three elements of the sound are clearly identifiable. Hereafter, however, the elements are followed by a very faint ‘bwoo’ sound, not present in the door sound (visible as a weak trace on the sonogram). The power spectrum of one of the elements is provided in figure 2*d*. The full recording contains 34 repetitions of this vocalization, which have an average inter-onset interval of about 3.2 s. Figure 2*e* shows three successive vocalizations, illustrating the repetitive nature of the vocalization.

#### (ii) Slamming door followed by speech-like mumble

**Figure 3.**
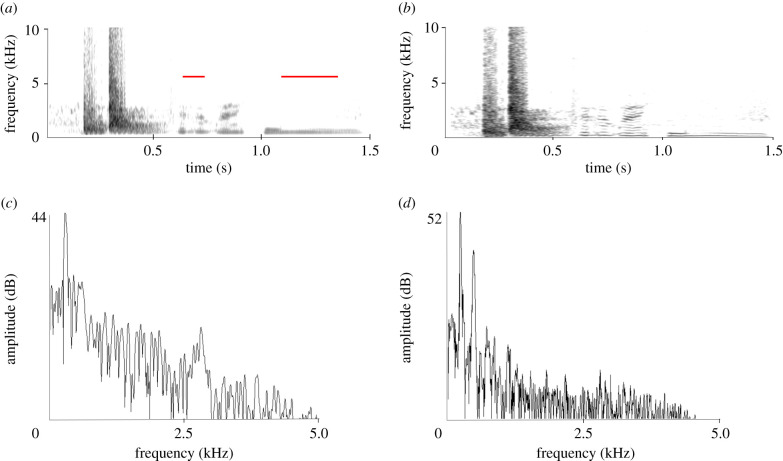
(*a*) Broadband and (*b*) narrowband sonograms of an imitated door sound, followed by a soft speech-like mumbling (electronic supplementary material, SI 3); (*c*) and (*d*) show power spectra of the parts indicated in red in (*a*).

Figure 3*a* shows a variant of the previous vocalization. The first two elements are similar to those of the slamming door recording, but instead of the third element Ripper produced a soft ‘mumbling’ sound consisting of two subsections, each at a low frequency (electronic supplementary material, SI 3). These sounds are voice-like to the human ear, but no clear words are discernable. The latter part has similarity to the faint ‘bwoo’ at the end of the vocalization discussed above, but is more clearly audible. The narrowband sonogram (figure 3*b*) and power spectra (figure 3*c*,*d*) for two parts of the mumbling sound reveal that the sounds are part of a harmonic spectrum with a fundamental of 270 Hz (figure 3*c*) and 225 Hz (figure 3*d*). The duration of the full sequence is comparable to that of the slamming door imitation (approximately 1.5 s). The full recording of this sound contains 10 repetitions, with a varying inter-onset interval with an average of 5.1 s.

#### (iii) You bloody foo..

**Figure 4.**
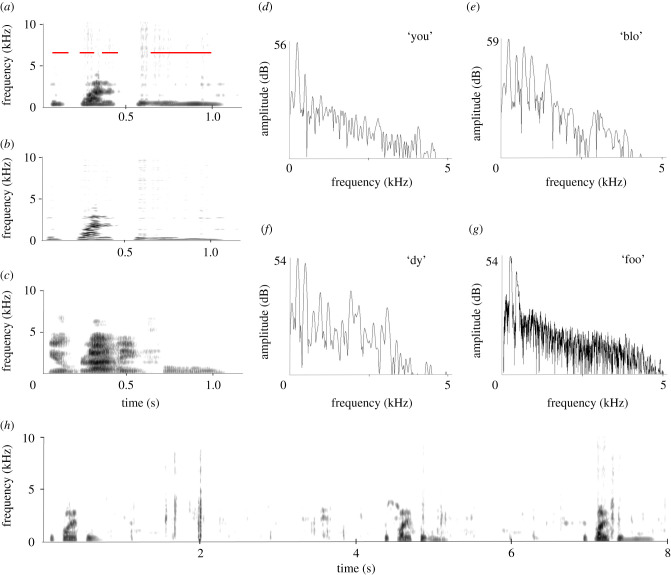
(*a*) Broadband and (*b*) narrowband sonograms of the ‘you bloody foo…’ sound produced by Ripper (electronic supplementary material, SI 4). (*c*) A human male voice producing the same utterence; (*d*)–(*g*) power spectra of the indicated parts (red) of Ripper's vocalization. Note the similarities in the fundamentals of the harmonic spectra. (*h*) Three subsequent vocalizations showing the stereotyped nature and regularity in interval duration.

This vocalization (figure 4*a*,*b*) bears the closest resemblance to a human voice, sounding like someone saying ‘you bloody foo’ (or ‘fool’, or ‘food’) (electronic supplementary material, SI 4). It was recorded when Ripper was on the inside and up against the wire fence, with the microphone alongside at less than a metre distance much of the time. PJ Fullagar was nearby because that was the way to enrage him into display, indicating Ripper was very human-oriented in his displays. Ripper would come up onto the narrow bank on the inside of the fence and scramble along ‘attacking’ anyone on the outside. He called repeatedly then dashed about on the small patch of water within the pen splashing water everywhere. The vocalization is most likely an imitation of a phrase he heard repeatedly from his caretaker, but it is not known at which age he was exposed to it. Figure 4*a* shows the broadband sonogram for the vocalization—for comparison figure 4*c* provides the sonogram of a male voice producing the same sequence (not the voice to which Ripper was exposed). Figure 4*b* is a narrowband sonogram, showing that this vocalization also originates from a harmonic spectrum. The whole vocalization consists of four discernible parts corresponding to ‘you’, ‘blo’, ‘dy’ and ‘foo’. Figure 4*d–g* provides the power spectra for these parts. They all show peaks in the lower frequency range indicating the presence of a fundamental of around 220 Hz (figure 4*d* 220 Hz; figure 4*e* 225 Hz; figure 4*f* 215 Hz; figure 4*g* 193 Hz) with harmonics. This illustrates that the different vowel-like sounds of the four components do not differ much in their fundamental, but differ in which parts of the harmonic spectrum are emphasized. The recording of this vocalization contains 10 repetitions with an average inter-onset interval of 4.2 s. A representative section of three successive vocalizations is shown in figure 4*h*.

### (b) Duck 2—whistle-kick vocalization and Pacific black duck imitation

**Figure 5.**
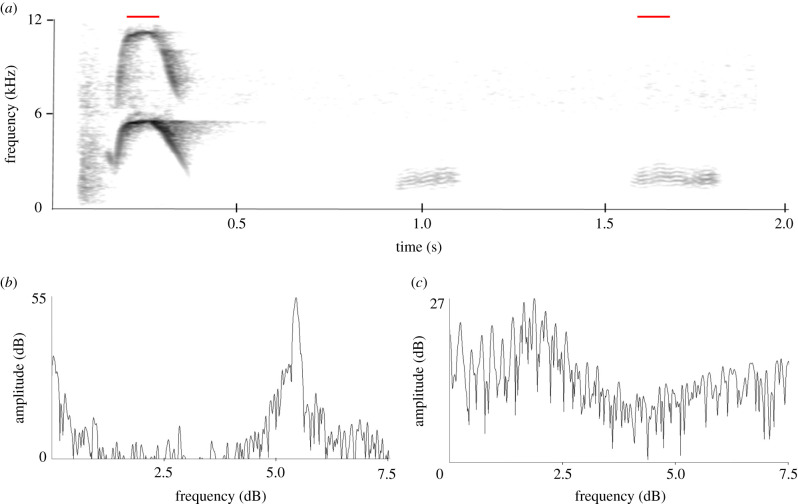
(*a*) Narrowband sonogram of a whistle-kick sound of duck 2 (electronic supplementary material, SI 5). The sound starts with a percussion-like sound, followed by a whistle and after that two imitations of a Pacific black-duck quack. Note the difference of the percussion sound and the whistle from the normal whistle-kick sound as shown in figure 1. (*b*) A power spectrum taken at the highest point of the whistle, indicated in red. (*c*) Power spectrum of a quack, showing a clear harmonic spectrum with a fundamental of 0.23 kHz.

Figure 5*a* shows a narrowband sonogram of a whistle-kick vocalization by this duck which has two black-duck quacks at the end. There are two recordings of this duck: a series of 72 and one of 45 vocalizations, several of which included 1–3 quacks (electronic supplementary material, SI 5). The first part of all vocalizations consists of a rather loud percussion sound, showing some resemblance to the door slamming sound produced by Ripper. This second duck had been exposed to Ripper, which may have affected this part of the sound. This sound is followed by a whistle. However, as has been reported for other captive-bred musk ducks [22], the whistle shape differs from that of wild musk ducks. Instead of beginning at a high frequency and then descending, as the normal whistles do (figure 1*a*), this whistle has an inverted U-shape, going up first and next dropping again quickly in frequency. The series of 72 vocalizations, given at a quite constant rate with an average inter-onset interval of 4.7 s, contains three vocalizations which add 1, 2 and 2, respectively, black-duck quacks at the end. In the second series, again produced at a regular pace with an average inter-onset interval of 4.6 s, 9 out of the 45 have 1–3 quacks added (1 quack – *n* = 3, 2 quacks - *n* = 5, 3 quacks - *n* = 1). In both series, there are switches to and from vocalizations with quacks. The presence of the quacks has no clear effect on the inter-onset interval. The sonogram (figure 5*a*) and the power spectra (figure 5*b,c*) show a remarkable difference in structure between whistles and quacks. While the whistle shows no indication of an underlying harmonic spectrum, this is clearly visible for the quacks. The quack power spectrum shows a fundamental of about 230 Hz, comparable to the values produced by Ripper when imitating the voice-like sounds. When recording this sound, PJ Fullagar noted that the beak of the musk duck was opening when producing the quacks.

## 4. Discussion

The results presented above show imitations of allospecific vocalizations by Ripper and a second musk duck, and of mechanical sounds by Ripper. In addition, these ducks might not have been the only captive-reared ones to imitate allospecific sounds. We received the following note (B Makins 2021, personal communication) about a male musk duck reared from an egg transferred to Pensthorpe (Norfolk, UK): ‘The male was a wonderful mimic when he was quite young you could hear a lot of coughing and a snorting pony which lived next door to him. He even tried a unpronounceable hello to the gardener’. Another observation concerns a male musk duck raised at Slimbridge Wildfowl Trust (UK) which was at least two years of age when he was observed to produce an imitation of the characteristic cough of his bird keeper and also of a squeak of a turnstile (M Lubbock 2021, personal communication). As far as known, these birds have not been recorded, and hence the observations cannot be confirmed independently. However, the data of the two individuals presented above, combined with the presence of vocal differences between the two geographically separated populations and reports that captive south-eastern ducks often substitute the sound preceding the whistle by an unstructured swoosh-like sound, have a differently shaped whistle and show other idiosyncratic deviant vocalizations considerably different from wild birds [20], indicate the vocal development of the musk duck fits the various criteria used as evidence for vocal production learning in songbirds, parrots and hummingbirds. This has implications for hypotheses concerning the evolution of vocal production learning in birds. One hypothesis is that vocal learning evolved once in the common ancestor of the taxa showing vocal learning, followed by subsequent losses in vocal non-learning taxa (e.g. [24]), while an alternative one is that it arose in various groups independently. The musk duck belongs to a basal clade in the avian phylogeny. Thus, if vocal learning evolved only once this must have been almost at the root of the avian tree with subsequent losses in many branches. We consider this less likely than the scenario of several independent origins. Below we discuss in more detail what the observations tell about the context and mechanisms of vocal learning and vocal production in the musk duck and also indicate some relevant life-history traits.

It is not known at what age Ripper was transferred to an outdoor pond but it is most likely that he may not have been exposed to the door sound for more than a few weeks, quite early in life. Hence, at least some of the sounds imitated by Ripper were acquired at an early age, but produced when adult, suggesting early memorization of a vocal model. This resembles template-based vocal learning as shown in songbirds. Unfortunately, it is not known at what age Ripper produced imitations for the first time, but this might have been after several years. The recordings were made at 4 years of age and a comment stated that the sounds were only recently noticed by the caretaker. Whether there is a sensitive phase for learning cannot be assessed from the current data. In the wild, musk ducks would easily hear males from the time of hatching, as males would be calling in the area. Females do not go far away to nest and young travel on the backs of the female or follow her when larger. Field observations on wild birds indicate that young males develop the normal vocalizations gradually, showing incomplete and more variable vocalizations [22]. Whether such developing vocalizations can still be affected by hearing adult males is unknown, although it is reported that young males attend to bouts of display activity performed by older males [19]. This may indicate that not just early, but also later experience has a role in vocal development, perhaps to adjust and fine-tune the memorized sounds. It is not known when the second male may have acquired the Pacific black-duck quacks. He was kept in a pond at which black ducks were present, but whether he had any specific interactions with these and at what age is unknown.

The imitations produced by both ducks contain distinct parts, which can originate from different models (door and human voice) as shown by Ripper, or may be a combination of sounds that develop in isolation (deviating whistle sounds are known from other isolation-reared musk ducks—[20]) and imitated ones (Pacific black duck), as shown by duck 2. It suggests that vocal imitations do not arise from copying a single sequence, but from putting together material obtained and memorized from various sources.

The deviating sounds are produced during repetitive visual displays, replacing the vocal elements that are present normally, but apparently not affecting the repetitive structure or the display context in which the vocalizations are produced—a phenomenon also observed in songbirds, in which element structure is more strongly affected by cross-fostering than the temporal structure of songs (e.g. [25]). The various vocalizations by both ducks maintained approximately similar inter-onset intervals (3–5 s) between separate calls to those of the *ca* 4 s [20] present in wild ducks, despite the sometimes longer duration of the deviant sounds.

The vocal development in musk ducks thus shows clear parallels to the vocal learning of, in particular, songbirds and parrots. Ripper's allospecific copying also shows striking parallels to some examples of vocal learning in mammals, most notably that of Hoover, an isolated hand-reared harbour seal, which later in life produced speech sounds showing resemblance to his caretaker [26], and of a captive Asian elephant, Koshik, which also imitated the speech sounds of his caretaker [9]. Many other cases of species and individuals for which imitation of human speech sounds or allospecific vocalizations have been reported are from isolated, hand-reared or cross-fostered species and are reported to result from extensive social interactions or strong bonds between the imitating individual and the model (e.g. [13]).

The three different sound imitations produced by Ripper were all accompanied by the visual whistle-kick posture. Whether visual elements of Ripper's display were identical when given with each of his three sounds is unknown. One of the recordings mentions that the kicks with the feet, which are normally part of the display, were lacking, but whether the display differed from that of wild musk ducks in other respects is unknown. It would demonstrate remarkable flexibility in vocal control if Ripper could use each of his sounds in exactly the same context, but it might well be that the different vocalizations relate to different levels of arousal, as has been mentioned for the visual displays [22]. So far, there are no analyses or reports in which the visual displays of other captive-reared musk ducks with vocal deviations have been examined.

Another interesting aspect of the vocalizations is how they are being produced. The power spectra of the soft percussion-like sound at the beginning of the normal whistle-kick shows three harmonics with a frequency of around 595 Hz. The power spectra of the speech-like elements present in Ripper's vocalizations and the quacks of duck 2 also show a harmonic spectrum, in this case one in which the overtones are subsequently modified in amplitude by an apparently adjustable vocal tract filter. At about 220 Hz, the fundamental frequency for these sounds is lower than the normal sound preceding the whistle. The sonograms and power spectra for these sounds bear close resemblance to those of human voices producing different vowels. In humans, the vocal chords give rise to a harmonic spectrum with fundamental frequencies between 80 and 300 Hz [27]. The configuration and modifications of the human vocal tract selectively filter or amplify particular frequency regions, giving rise to so-called formants. The combination of different formants affects which vowel is being produced. Ripper's vowel-like sounds also seem the result of selective filtering, as suggested by comparing the power spectra for the various parts of the ‘you bloody foo’ phrase. They all have the same underlying harmonic structure, but differ in the emphasized frequency bands. Such structures are similar to those of several other birds for which vowel imitating spectra have been analysed in more detail, such as the grey parrot and yellow-naped Amazon [16] and the hill mynah [11]. As the anatomy of the musk duck vocal tract has not been described, it is not clear which structures might be involved in the vocal tract filtering. However, one parameter known to adjust resonance features of the harmonic spectrum of anserine calls is the beak opening [28]. Beak opening also accompanied the production of the quacks produced by duck 2. The sonograms of the whistle structures suggest there may also be other mechanisms involved in sound production. Studies of the syrinx of other Anatidae show complex and also asymmetrical morphologies (e.g. [29]). Different ways of vocalizing have also been suggested for mallard, which include quacks very similar in sonographic structure to the musk duck imitations of the Pacific black duck (e.g. [29,30]). Although sonograms cannot present unambiguous evidence about the underlying mechanical origin of the sound signal (e.g. [31]), the structure of the vocalizations indicates the presence of a flexible and sophisticated control over vocal production. Another open question concerns the neural structure(s) involved in producing the imitations. In a comparative study on brain size in waterfowl, Iwaniuk & Nelson [32] note that musk duck brain size is relatively large. Unfortunately, it is not known which areas are responsible for the brain size difference and whether this includes any areas potentially involved in learning or producing vocalizations, such as the telencephalon. This area, which in songbirds and parrots contains nuclei involved in vocal learning, is relatively large in these groups compared to other avian taxa, but, interestingly, it is also relatively large in waterfowl [33].

As far as is known, vocal learning is not present in any other Anserine species, so what makes the musk duck special? One relevant feature might be that musk ducks are more altricial than other Anserine species [18], resulting in longer social contact with their mothers compared to other Anserine species. Musk ducks produce only a few offspring at a time, which rely on maternal feeding until almost fully grown [18]. They are, like Anserines in general, likely to show filial and sexual imprinting. As a result, an isolated hand-reared musk duck, such as Ripper, most likely forms a strong attachment to a human caretaker. Being altricial, the longer period of dependency might also be accompanied by a more gradual development of neural systems, providing the scope for a larger impact of experience (learning) on behavioural development. Unfortunately, it is still unclear which species are the closest relatives of musk ducks [34] and thus how musk duck vocalizations and their development compares to that of these relatives. Also, while the conditions that may contribute to vocal learning may be present in musk ducks, why it evolved in this species and not in other Anserines is not clear. Of relevance might be that the musk duck is a lekking species and a further study of whether leks show variation in vocal characteristics might be useful, in particular as young males attend to displaying older birds [21].

To conclude, the Australian musk duck demonstrates an unexpected and impressive ability for vocal learning. The findings presented here call for a more extensive and systematic study of this and related, or other, species belonging to this clade and demonstrate the need and usefulness for a much wider and more systematic search for examples of vocal learning among avian taxa to provide more extensive material for comparative studies of vocal learning (see also [10]). This may contribute important insights into the behavioural and neural mechanisms involved, and also into the vocal production mechanisms that enable the production of such sounds. In combination with analysing ecological, social, phylogenetic and life-history parameters, this will provide a basis for understanding why and how vocal production learning has evolved in certain species or groups and not in others.

## Data accessibility

Fragments of the audio files are provided as electronic supplementary material [35]. Full recordings of the sounds produced by Ripper and analysed for this paper are deposited at the Australian National Wildlife Collection as files X49184 and X49185 and are also available from the authors, as is the full recording of duck 2.
